# Efficacy of transcranial magnetic stimulation in the treatment of combat-related PTSD: a systematic review and meta-analysis

**DOI:** 10.3389/fpsyt.2026.1756576

**Published:** 2026-03-05

**Authors:** Hesed Virto-Farfan, Fritz Fidel Váscones-Román, Valeria Rivera, Olga Karpenko, Elena Bochkina, Ekaterina Parshakova, Alexey Sinev, Gustavo E. Tafet, Niels Pacheco-Barrios

**Affiliations:** 1 Neuroscience Research Center (CENEURO), DGI, Andean University of Cusco, Cusco, Peru; 2 Facultad de Medicina Humana, Universidad Peruana Cayetano Heredia, Lima, Peru; 3 Facultad de Medicina Humana, Universidad de San Martín de Porres, Lima, Peru; 4 Mental-Health Clinic No.1 named after N.A.Alexeev, Moscow, Russia; 5 Department of Psychiatry and Behavioral Sciences, Texas A&M University, College Station, TX, United States; 6 Carrera de Medicina Humana, Universidad Científica del Sur, Lima, Peru; 7 Department of Neurosurgery, Brigham and Women’s Hospital, Boston, MA, United States

**Keywords:** combat-related PTSD, neuromodulation, systematic review and meta-analysis, transcranial magnetic stimulation, veterans, TMS

## Abstract

**Introduction:**

Combat-related post-traumatic stress disorder (PTSD) remains highly prevalent among military personnel and veterans and is frequently chronic, disabling, and only partially responsive to first-line pharmacological and psychotherapeutic interventions. Given the central role of fronto-limbic circuit dysfunction in PTSD, transcranial magnetic stimulation (TMS) has emerged as a biologically plausible neuromodulatory strategy, yet its protocol-level efficacy in combat-exposed populations is not well established. Clarifying whether specific TMS modalities offer clinically meaningful benefit beyond sham, and whether any protocol can be prioritized, is critical for rationally integrating TMS into veteran-focused care pathways.

**Methods:**

This systematic review and meta-analysis followed PRISMA 2020 and Cochrane Handbook recommendations and was prospectively registered in PROSPERO (CRD420251105555). We searched PubMed, SCOPUS, Embase, Web of Science, and EBSCO (March–June 2025) for clinical studies of adults with combat-related PTSD (DSM-IV, DSM-5, ICD-10, or ICD-11) receiving any TMS modality (rTMS, theta-burst stimulation, deep TMS, synchronized or accelerated TMS), compared with sham, standard care, or both. Primary outcomes were changes in PTSD severity measured with validated instruments (e.g., CAPS, PCL-5); secondary outcomes included depressive and anxiety symptoms, psychosocial functioning, acceptability, and safety. Random-effects meta-analyses (DerSimonian–Laird) were conducted for within-group pre–post change and between-group mean differences (TMS vs. control); heterogeneity was quantified with I². Risk of bias in randomized trials was assessed using the Cochrane RoB 2.0 tool.

**Results:**

From 191 records, 7 studies (n = 963) were included in the quantitative synthesis. Five studies contributed pre–post data (including one of the randomized controlled trial that presented the pre and post data of the TMS group), showing a large, clinically meaningful pooled reduction in PTSD symptoms after TMS (pooled mean change −20.39 points; 95% CI −23.94 to −16.83; p < 0.001; I² = 88.7), with the greatest improvements observed in high-frequency (10 Hz) left DLPFC rTMS protocols delivered over 20–30 sessions. In contrast, three randomized controlled trials (n = 116) comparing active TMS with sham yielded a non-significant pooled mean difference favoring TMS (MD −3.83; 95% CI −16.32 to 8.65; p = 0.098; I² = 56.9), suggesting that a substantial portion of symptom improvement may reflect non-specific or shared therapeutic factors. Subgroup analyses hinted at benefit for conventional rTMS and inconclusive effects for deep TMS, but were underpowered and did not identify any modality as clearly superior. Across studies, TMS was well tolerated: no serious adverse events were reported, dropout rates were low (~7%), and adverse effects were predominantly mild (transient headache, scalp discomfort, fatigue). Overall, the evidence indicates that TMS yields robust within-group clinical improvement and an excellent safety profile in combat-related PTSD, while the specific advantage over sham and the comparative superiority of individual TMS protocols remain uncertain, underscoring the need for larger, protocol-focused randomized trials with standardized parameters and longer follow-up.

**Systematic Review Registration:**

https://www.crd.york.ac.uk/PROSPERO/view/CRD420251105555 **, identifier CRD420251105555.**

## Introduction

Post-traumatic stress disorder (PTSD) is a mental disorder that develops as a result of powerful psychotraumatic exposure of a threatening or catastrophic nature, accompanied by extreme stress and characterized by the repeated experience of the traumatic event in the present time in the form of vivid intrusive memories accompanied by fear or terror, hypervigilance, flashbacks, or nightmares, as well as avoidance behavior. The average prevalence rate of PTSD among war veterans is 15–30% and varies depending on the socioeconomic status of the country, the nature of the conflict, research methodology, as well as gender and age (1, 2).

Another feature of PTSD is its high comorbidity with mental disorders such as substance abuse, depression, and anxiety disorders, leading to reduced social functioning, impaired quality of life, and a high suicide risk (3–5). Thus, the development of effective and accessible interventions for the treatment of PTSD is an important task for improving public health.

First-line treatment options for PTSD include psychopharmacology and psychotherapeutic approaches. Currently, the FDA has approved only two selective serotonin reuptake inhibitor (SSRI) antidepressants, sertraline and paroxetine (6) as well as one serotonin-norepinephrine reuptake inhibitor (SNRI) antidepressant, venlafaxine, though its use is limited. Many patients show insufficient response to antidepressant therapy, as these drugs do not affect the fundamental mechanisms of PTSD pathogenesis (7). The delayed onset of their clinical effect is often extremely difficult for patients to tolerate, sharply reducing compliance. Patients with PTSD frequently discontinue treatment prematurely on their own (7, 8).

Among non-pharmacological treatment methods, Eye Movement Desensitization and Reprocessing (EMDR) is widely used a psychotherapy method developed by Francine Shapiro in 1987 for the treatment of post-traumatic stress disorder. In 2013, it was recognized by the WHO (2013) and the American Psychological Association (APA) as having “Level A evidence” for PTSD treatment (9). The method is based on bilateral brain stimulation (eye movements, tactile or auditory signals), which facilitates the reprocessing of traumatic memories. The neurobiological mechanisms of EMDR involve reducing amygdala hyperactivity, normalizing hippocampal and prefrontal cortex function, and restoring the balance between the parasympathetic and sympathetic nervous systems (10).

In recent years, researchers have focused on alternative pathophysiological mechanisms of PTSD, including dysfunction of the hypothalamic-pituitary-adrenal (HPA) axis, glutamatergic transmission, and the endocannabinoid system, as well as neuropeptide Y and oxytocin (11).

Cannabidiol (CBD) demonstrates potential efficacy in PTSD treatment by modulating the endocannabinoid system (CB1 and CB2 receptors), influencing serotonin (5-HT1A) receptors, and exerting anti-inflammatory effects, as supported by preclinical animal studies (12). Clinical studies in humans are limited but suggest that CBD may reduce anxiety and improve fear extinction, while synthetic cannabinoids (nabilone) reduce nightmares in PTSD patients. Despite its favorable safety profile, legal restrictions hinder further research.

Oxytocin is both a neuropeptide and peptide hormone produced by hypothalamic nuclei and may influence PTSD symptoms by improving social perception, emotional regulation, and attention (11). It also affects amygdala interactions with other brain structures, though its effects depend on sex and dosage (13). Neuropeptide Y (NPY) has anxiolytic potential but requires further research to confirm its therapeutic role. Thus, while oxytocin and other neuropeptides open new therapeutic possibilities, their use requires an individualized approach and additional clinical trials.

The primary changes in PTSD affect the amygdala–hippocampus–medial prefrontal cortex circuit, which is responsible for fear learning, threat regulation, and emotional control. Dysfunctions in these areas lead to heightened fear, difficulty in fear extinction, and impaired emotional regulation (14). PTSD patients often exhibit altered cortisol and adrenocorticotropic hormone (ACTH) levels. Some studies report reduced cortisol levels and increased feedback sensitivity, which may further decrease cortisol levels (15).

Modern therapeutic practice and research question the effectiveness of treatment for PTSD patients, particularly combat-related PTSD (16, 17), prompting interest in alternative methods such as transcranial magnetic stimulation (TMS). By directly targeting key neurophysiological mechanisms of the disorder, TMS can modulate prefrontal cortex function, which is hypoactive in PTSD, thereby counteracting amygdala hyperreactivity (18). TMS is a non-invasive treatment method involving magnetic impulses delivered at varying frequencies and intensities. Low-frequency stimulation (<5 Hz) has inhibitory effects, while high-frequency stimulation (≥5 Hz) has excitatory effects on brain activity.

Transcranial magnetic stimulation (TMS) is a non-invasive neuromodulation technique in which rapidly changing magnetic fields, generated by a coil placed over the scalp, induce focal electric currents in underlying cortical tissue, thereby modulating neuronal excitability and synaptic activity. Depending on stimulation parameters such as frequency, intensity, and pattern, TMS can either enhance or inhibit cortical activity and influence interconnected brain networks. In psychiatric practice, TMS has been increasingly adopted as a therapeutic tool, and its relevance for post-traumatic stress disorder (PTSD) is supported by neurocircuitry models describing impaired top-down regulation of limbic fear and stress networks by prefrontal cortical regions (19, 20). Within this framework, stimulation of prefrontal targets—most commonly the dorsolateral or medial prefrontal cortex—has been proposed to strengthen regulatory control over hyperresponsive threat-processing circuits and facilitate fear extinction processes (19, 20). Early meta-analytic evidence and subsequent network meta-analyses suggest that repetitive TMS applied to prefrontal regions may result in clinically meaningful reductions in PTSD symptom severity, although treatment effects appear sensitive to stimulation parameters and clinical heterogeneity (21, 22). More recent randomized trials indicate that intermittent theta-burst stimulation, a time-efficient patterned form of TMS designed to mimic endogenous theta–gamma coupling, may offer comparable therapeutic benefits (23). Nevertheless, systematic evaluations using Cochrane methodology emphasize variability across studies and highlight the need for cautious interpretation and continued optimization of stimulation protocols within psychiatric populations (24).

TMS therapy varies depending on the number and duration of magnetic impulses delivered. Repetitive TMS (rTMS) rapidly delivers magnetic impulses to the brain at regular intervals and is the most widely studied and commonly used type of TMS. Recently, new forms of TMS have emerged, such as theta-burst stimulation (iTBS), synchronized TMS (sTMS), and EEG-guided TMS. Theta-burst TMS operates at ~50 Hz and lasts ~3 minutes, whereas rTMS operates at ~5–20 Hz and requires ~20–30 minutes per session. Synchronized TMS aligns magnetic fields with an individual’s intrinsic alpha frequency, potentially enhancing therapeutic effects. EEG-guided TMS involves placing a TMS coil over an EEG apparatus to measure brain activity during stimulation, allowing real-time optimization of TMS parameters.

The U.S. Food and Drug Administration (FDA) has approved TMS for the treatment of obsessive-compulsive disorder (OCD). As an adjunctive therapy for pharmacoresistant OCD, TMS has a Level 2 evidence grade and a recommendation strength of B (25, 26). Patients with PTSD are recommended to undergo repetitive transcranial magnetic stimulation (27).

The current meta-analysis aims to compare the efficacy of different TMS protocols, as most studies have focused on rTMS, while only a few have explored newer forms (iTBS, synchronized TMS, or EEG-guided TMS). It is important to note that TMS efficacy may vary depending on coil type and placement, stimulation parameters, and patient-specific factors such as sex and age, with many studies featuring heterogeneous patient groups (28). Although numerous randomized controlled trials (RCTs) have been conducted, most had small sample sizes and limited statistical power to detect differences between TMS protocols. Long-term effects of TMS remain unclear, and there is a lack of studies specifically involving combat veterans.

This systematic review and meta-analysis focuses specifically on military personnel and veterans with combat-related PTSD. This population-level restriction was defined *a priori* to reduce trauma-related heterogeneity and enhance internal validity, as combat-related PTSD typically results from prolonged, cumulative, and repeated exposure to life-threatening stressors inherent to combat environments, rather than from isolated or single-event trauma. Such exposures occur within a relatively uniform occupational and contextual framework, which confers greater clinical and neurobiological homogeneity. Military and veteran populations are also characterized by high rates of chronic PTSD, frequent psychiatric comorbidities, and a substantial burden of treatment resistance, contributing to a persistent therapeutic gap. By restricting the synthesis to combat-related PTSD, this review seeks to generate clinically meaningful and methodologically robust estimates of the efficacy and safety of transcranial magnetic stimulation in a population for whom neuromodulation-based interventions may be particularly relevant and for whom effective treatment options remain limited.

## Methods

This systematic review and meta-analysis followed the guidelines established by the Cochrane Handbook and the Preferred Reporting Items for Systematic Reviews and MetaAnalyses (PRISMA) 2020 statement (29, 30) The protocol was prospectively registered on PROSPERO, under number: CRD420251105555.

### Search strategy and data extraction

The research presented in this systematic review and metaanalysis followed the PRISMA guidelines (31). We searched the PubMed, SCOPUS, Embase, Web of Science and EBSCO databases from March to June 2025. The following terms were used, combined with Boolean operators (“AND” and “OR”): PubMed: (“Transcranial Magnetic Stimulation”[Mesh] OR “Transcranial Magnetic Stimulation”[Title/Abstract] OR “TMS”[Title/Abstract] OR “rTMS”[Title/Abstract] OR “theta burst stimulation”[Title/Abstract] OR “TBS”[Title/Abstract] OR “deep TMS”[Title/Abstract] OR “dTMS”[Title/Abstract] OR “synchronized TMS”[Title/Abstract] OR “sTMS”[Title/Abstract]) AND (“Stress Disorders, Post-Traumatic”[Mesh] OR “Post-Traumatic Stress Disorder”[Title/Abstract] OR “PTSD”[Title/Abstract] OR “combat PTSD”[Title/Abstract] OR “military PTSD”[Title/Abstract] OR “war-related PTSD”[Title/Abstract]) AND (“Military Personnel”[Mesh] OR “Veterans”[Mesh] OR veteran*[Title/Abstract] OR soldier*[Title/Abstract] OR military[Title/Abstract] OR “armed forces”[Title/Abstract] OR combatant*[Title/Abstract]). SCOPUS: TITLE-ABS-KEY ((“Transcranial Magnetic Stimulation” OR “TMS” OR “rTMS” OR “theta burst stimulation” OR “TBS” OR “deep TMS” OR “dTMS” OR “synchronized TMS” OR “sTMS”) AND (“Post-Traumatic Stress Disorder” OR “PTSD” OR “combat PTSD” OR “combat-related PTSD” OR “military PTSD” OR “war-related PTSD”) AND (veteran* OR soldier* OR military OR “armed forces” OR combatant*)). Embase: (‘transcranial magnetic stimulation’/exp OR ‘transcranial magnetic stimulation’:ti,ab OR ‘TMS’:ti,ab OR ‘rTMS’:ti,ab OR ‘theta burst stimulation’:ti,ab OR ‘TBS’:ti,ab OR ‘deep TMS’:ti,ab OR ‘dTMS’:ti,ab OR ‘synchronized TMS’:ti,ab OR ‘sTMS’:ti,ab) AND (‘posttraumatic stress disorder’/exp OR ‘PTSD’:ti,ab OR ‘combat PTSD’:ti,ab OR ‘combat-related PTSD’:ti,ab OR ‘war-related PTSD’:ti,ab OR ‘military PTSD’:ti,ab) AND (‘military personnel’/exp OR ‘veteran’/exp OR veteran*:ti,ab OR soldier*:ti,ab OR military:ti,ab OR ‘armed forces’:ti,ab OR combatant*:ti,ab). Web of Science (Clarivate): TS=(“Transcranial Magnetic Stimulation” OR “TMS” OR “rTMS” OR “theta burst stimulation” OR “TBS” OR “deep TMS” OR “dTMS” OR “synchronized TMS” OR “sTMS”) AND TS=(“Post-Traumatic Stress Disorder” OR “PTSD” OR “combat PTSD” OR “combat-related PTSD” OR “military PTSD” OR “war-related PTSD”) AND TS=(veteran* OR soldier* OR military OR “armed forces” OR combatant*). EBSCO: (TMS OR “Transcranial Magnetic Stimulation” OR rTMS OR “theta burst stimulation” OR TBS OR “deep TMS” OR dTMS OR “synchronized TMS” OR sTMS) AND (PTSD OR “Post-Traumatic Stress Disorder” OR “combat PTSD” OR “combat-related PTSD” OR “military PTSD” OR “war-related PTSD”) AND (veteran* OR soldier* OR military OR “armed forces” OR combatant*).

### Eligibility criteria Inclusion criteria

The inclusion criteria were studies involving military personnel or veterans diagnosed with combat-related post-traumatic stress disorder (PTSD) according to DSM-IV, DSM-5, ICD-10, or ICD-11 criteria; treated with any form of transcranial magnetic stimulation (TMS), including repetitive TMS (rTMS), theta burst stimulation (TBS), deep TMS (dTMS), synchronized TMS (sTMS), or accelerated TMS (aTMS); compared with sham TMS, placebo, standard care (psychotherapy or pharmacotherapy), or combinations thereof; and reporting outcomes such as PTSD symptom reduction measured using *a priori* defined, validated PTSD-specific instruments, namely the Clinician-Administered PTSD Scale (CAPS; DSM-IV or DSM-5 versions) and the PTSD Checklist for DSM-5 (PCL-5). Studies employing non-validated or non-PTSD–specific outcome measures were excluded. Secondary outcomes included sustained efficacy at follow-up, depressive or anxiety symptoms, psychosocial functioning, acceptability (dropout rates), and safety (adverse events). The eligible study designs were randomized controlled trials (RCTs), controlled clinical trials, open-label trials, systematic reviews, and meta-analyses published in any language in peer-reviewed indexed journals.

The exclusion criteria were studies involving exclusively civilian or non-combat-related PTSD populations; animal studies, theoretical reviews, opinion papers, or case studies; studies without clearly defined PTSD diagnostic criteria; and those lacking a sham or control group comparison.

### Selection studies

All retrieved records were uploaded to the Covidence platform (https://app.covidence.org/) for systematic management of the screening process. Titles and abstracts were independently screened by two reviewers (HVF and FFVR), and records that did not directly address the research question or were identified as duplicates were excluded at this stage. Full-text articles were then independently assessed for eligibility by the same reviewer pair to ensure consistency in study selection. Data extraction was conducted independently by two reviewers (HVF and FFVR) using a predefined extraction form. Risk-of-bias assessment was performed independently by reviewers with methodological expertise (VR, OK, EB, EP), applying the appropriate Cochrane tools. Any discrepancies arising at any stage of the screening, extraction, or risk-of-bias assessment processes were resolved through discussion and consensus, and when necessary, consultation with a third reviewer (NPB). The use of a dedicated screening platform ensured reproducibility, traceability, and transparent documentation throughout all phases of the review process.

### Data extraction

The following data were extracted from the selected articles: authors, year of publication, study location, type of study, sample size and age, patient characteristics, duration of intervention, therapeutic scheme, follow-up time, main variables and main results. Two reviewers were responsible for extracting and managing the data independently, which were inserted into an Excel (Microsoft Corp.) spreadsheet; doubts were clarified with the help of the third researcher.

### Endpoints and sensitivity analyses

The endpoints of interest included PTSD symptom reduction, sustained efficacy during follow-up, depressive and anxiety symptom improvement, psychosocial functioning, treatment acceptability, and safety outcomes. PTSD symptom reduction was defined as a statistically significant decrease in total scores on validated instruments such as the Clinician-Administered PTSD Scale (CAPS) or the PTSD Checklist for DSM-5 (PCL-5). Sustained efficacy referred to the maintenance of clinical improvement throughout follow-up assessments. Safety outcomes included the frequency and severity of adverse events, such as headache, scalp discomfort, fatigue, or dizziness. Acceptability was assessed through treatment adherence and dropout rates. Sensitivity analyses were planned to evaluate the robustness of the pooled results by excluding studies at high risk of bias, those with small sample sizes, or those employing non-standard TMS parameters or protocols.

### Quality assessment

The risk of bias for each included study was evaluated using the revised Cochrane Risk of Bias (RoB2) tool for randomized trials (32) and the Evidence-Based Criteria Assessment (ECAs) (33) which categorizes studies as having a high, low, or unclear risk of bias. Two authors independently performed this assessment, and any disagreements were resolved by consensus with a third author.

### Statistical analysis

All statistical analyses were performed using R version 4.3.2 (R Foundation for Statistical Computing, Vienna, Austria) and the meta package. A random-effects model (DerSimonian–Laird method) was applied to account for inter-study variability. For within-group analyses, pooled mean changes in PTSD symptom scores (pre- to post-intervention) were calculated using the raw mean change (MRAW) as the effect size, with 95% confidence intervals (CIs). For between-group analyses, mean differences (MDs) between TMS and control (sham or standard therapy) groups were pooled using random-effects meta-analysis, converting standard errors to standard deviations when necessary. TMS and control (sham or standard Heterogeneity was quantified using the I² statistic, with values of 25%, 50%, and 75% representing low, moderate, and high heterogeneity, respectively. Sensitivity analyses were conducted using a leave-one-out approach to assess the influence of individual studies on the pooled effect, and subgroup analyses were performed according to the TMS protocol (rTMS, dTMS, or iTBS). Funnel plots and Egger’s regression test were used to explore potential publication bias when ≥10 studies were available. Forest plots were generated with RevMan 5.4 and R to visualize study-specific and pooled estimates. A two-tailed p value <0.05 was considered statistically significant.

## Results

### Study selection

**Figure 1:**
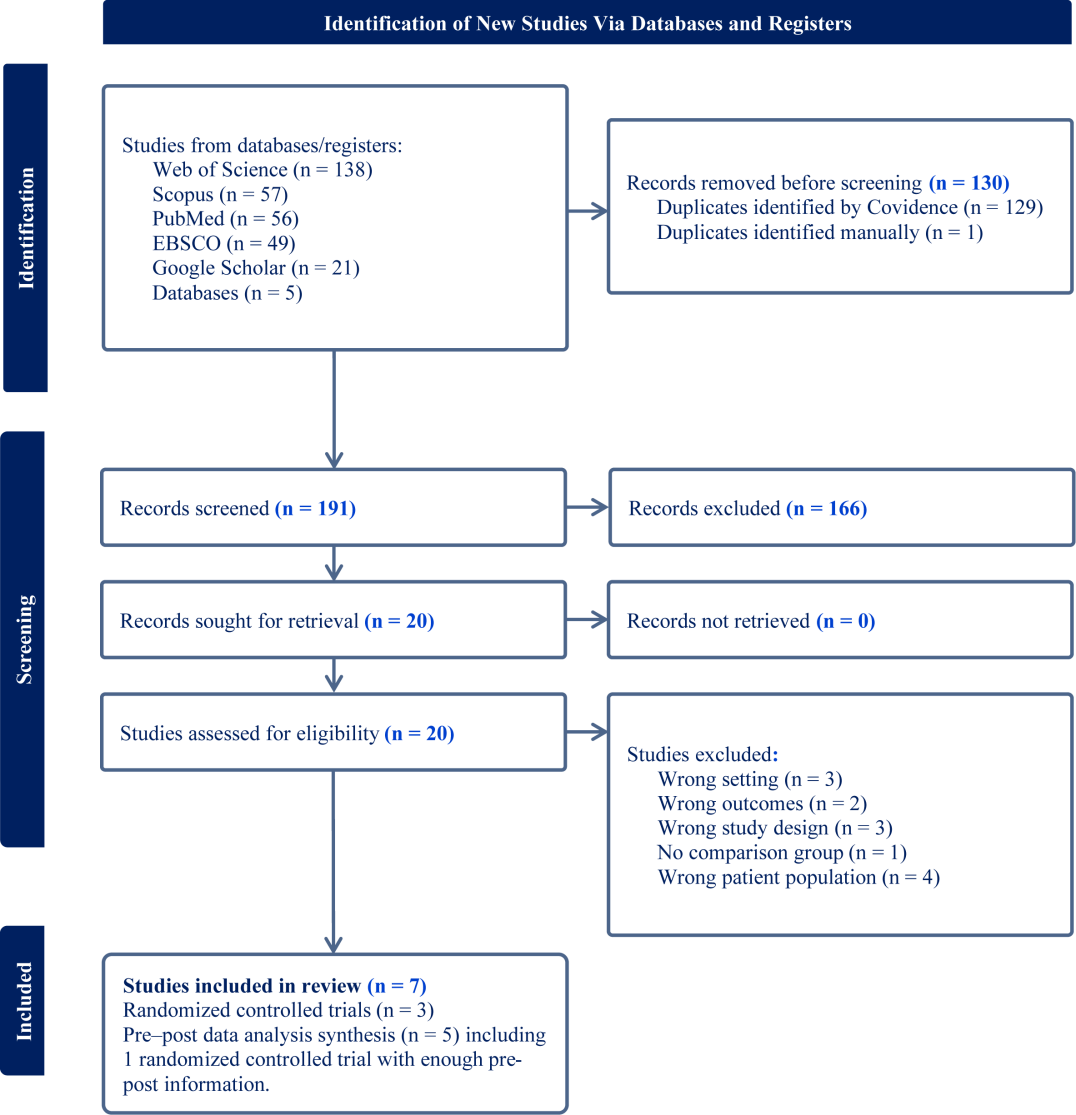
PRISMA 2020 flow diagram of the study selection process.

A total of 191 records were identified across PubMed, Scopus, Web of Science, EBSCO, and Google Scholar. After duplicate removal and screening, 20 full-text articles were assessed for eligibility; of these, 7 studies were included in the quantitative synthesis (meta-analysis) (**Figure 1**), due to the one group and control group analysis. The seven studies included in the meta-analysis (n = 963) provided sufficient quantitative data. Reasons for exclusion included non-combat PTSD populations (n = 4), absence of validated outcome measures (n = 3), non-comparative designs (n = 3), incomplete numerical data (n = 2), and duplicate cohorts (n = 1).

### Study characteristics

The studies were conducted between 2017 and 2023 across the United States, Turkey, Iran, Australia, involving a total of 963 participants across all included studies, predominantly male combat veterans aged 32–52 years. PTSD was diagnosed according to DSM-IV or DSM-5 criteria in all studies.

Repetitive transcranial magnetic stimulation (rTMS) was the most common modality, typically applied to the left dorsolateral prefrontal cortex (DLPFC) at 10 Hz and 120% of the resting motor threshold (RMT), delivered over 20–30 sessions within 4–6 weeks. Alternative modalities included deep TMS (dTMS) and intermittent theta-burst stimulation (iTBS). Comparators were primarily sham stimulation or standard pharmacotherapy. PTSD severity was measured with validated scales, most frequently the Clinician-Administered PTSD Scale (CAPS) and the PTSD Checklist for DSM-5 (PCL-5) (**Table 1**).

**Table 1:** Characteristics of studies included in the meta-analysis.

Author (year)	Country	Study design	N (total)	PTSD diagnosis	TMS type	Target site	Frequency (Hz)	Intensity (% RMT)	Sessions (n)	Comparator	PTSD scale	Baseline mean ± SD	Post-treatment mean ± SD	Mean change (Δ)	Follow-up (weeks)
Makale et al. (2023) (34)	Turkey	Open-label	195	DSM-5	rTMS	Left DLPFC	10	120	20	None	PCL-5	68.4 ± 12.7	47.8 ± 10.5	−20.6 ± 16.6	6
Philip et al. (2023) (35)	USA	RCT	658	DSM-5	rTMS	Left DLPFC	10	120	30	Sham TMS	PCL-5	73.2 ± 9.8	51.3 ± 11.5	−21.9 ± 1.5	8
Nursey et al. (2020) (36)	Australia	Pilot study	8	DSM-5	iTBS	Bilateral DLPFC	50 (burst)	80	10	None	PCL-5	60.1 ± 8.2	49.7 ± 7.5	−10.4 ± 5.8	3
Fryml et al. (2019) (37)	USA	RCT	8	DSM-5	dTMS	Medial PFC	20	120	15	Sham TMS	PCL-5	75.1 ± 12.0	58.1 ± 15.4	−17.0 ± 22.2	4
Kozel et al. (2019) (38)	USA	RCT	46	DSM-5	rTMS	Left DLPFC	10	120	20	Sham TMS	CAPS-5	74.4 ± 11.2	72.9 ± 12.6	−1.5 ± 5.3	4
Kozel et al. (2018) (39)	USA	RCT	32	DSM-5	rTMS	Left DLPFC	10	120	20	Sham TMS	CAPS-5	78.3 ± 10.5	54.2 ± 13.8	−24.4 ± 18.8	4
Ahmadizadeh et al. (2018) (40)	Iran	Open-label	16	DSM-IV	rTMS	Right DLPFC	1	100	15	None	CAPS	76.1 ± 9.1	50.7 ± 8.7	−25.5 ± 9.0	4

rTMS, repetitive transcranial magnetic stimulation; dTMS, deep transcranial magnetic stimulation; iTBS, intermittent theta-burst stimulation; DLPFC, dorsolateral prefrontal cortex; PFC, prefrontal cortex; RMT, resting motor threshold; CAPS, Clinician-Administered PTSD Scale; PCL-5, PTSD Checklist for DSM-5; MD, mean difference; dTMS, deep transcranial magnetic stimulation; iTBS, intermittent theta-burst stimulation; DLPFC, dorsolateral prefrontal cortex.

### Within-group analysis

**Figure 2:**
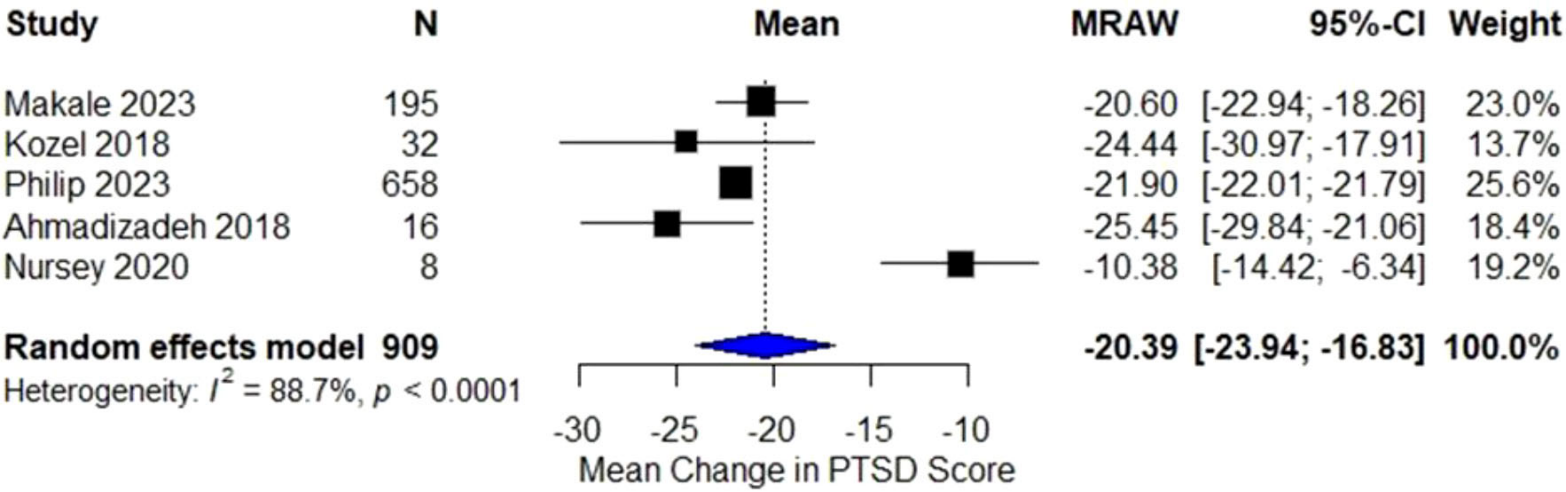
Forest plot of within-group analysis showing mean change in PTSD symptom scores before and after TMS treatment. The pooled mean change (MRAW) was −20.39 (95% CI [−23.94, −16.83]; p < 0.001) using a random-effects model (I² = 88.7%), indicating significant within-group improvement across studies.

Five studies reported pre–post data on PTSD symptom severity. The pooled within-group analysis demonstrated a significant reduction in PTSD symptoms following TMS (pooled mean change = −20.39 [95% CI −23.94 to −16.83]; p < 0.001; I² = 88.7%), indicating a robust clinical benefit (**Figure 2**).

All included studies showed improvement, with the largest changes reported in Ahmadizadeh 2018, Makale 2023, and Philip 2023. Subgroup inspection suggested that left-sided, high-frequency rTMS produced the greatest reductions in PTSD symptom scores, while right-sided or low-frequency protocols yielded smaller and more variable effects.

### Between-group analysis

**Figure 3:**
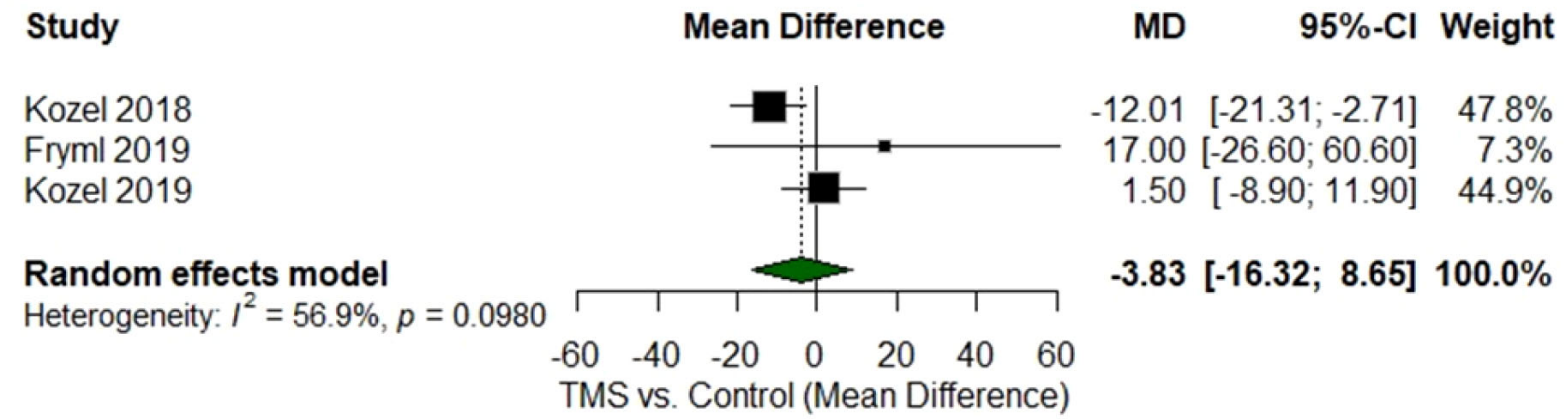
Forest plot of between-group comparison (TMS vs. sham/control).

Three randomized controlled trials compared TMS with sham stimulation. The pooled mean difference favored TMS but did not reach statistical significance (MD = −3.83 [95% CI −16.32 to 8.65]; p = 0.098; I² = 56.9%) (**Figure 3**). Subgroup analysis indicated a significant benefit for rTMS (MD = −12.0 [95% CI −21.3 to −2.7]; p < 0.05) compared with no effect for dTMS (MD = +17.0 [95% CI −26.6 to 60.6]). Sensitivity analyses using the leave-one-out method confirmed that no single study significantly altered the pooled estimates, supporting the robustness of the results (**Table 2**).

**Table 2:** Quantitative synthesis of TMS efficacy in combat-related PTSD.

Analysis	Included studies (n)	Total participants	Model	Pooled effect (95% CI)	p-value	I² (%)	Direction of effect
Within-group (pre–post)	5	909	Random effects	−20.39 [−23.94, −16.83]	<0.001	88.7	↓ CAPS/PCL-5
Between-group (TMS vs. control)	3	116	Random effects	−3.83 [−16.32, 8.65]	0.098	56.9	NS
Subgroup: rTMS only	2	78	Random effects	−7.31 [−20.6, 5.9]	0.24	48	↓
Subgroup: dTMS	1	8	Fixed effect	+17.0 [−26.6, 60.6]	0.45	–	↑
Sensitivity (leave-one-out)	–	–	Random	Stable estimates	–	–	–

Presents pooled mean changes and mean differences (95% CIs) for within-group and between-group analyses. Includes subgroup and sensitivity analyses evaluating the robustness of pooled results and differences by TMS modality (rTMS, dTMS).

### Risk of bias assessment

**Figure 4:**
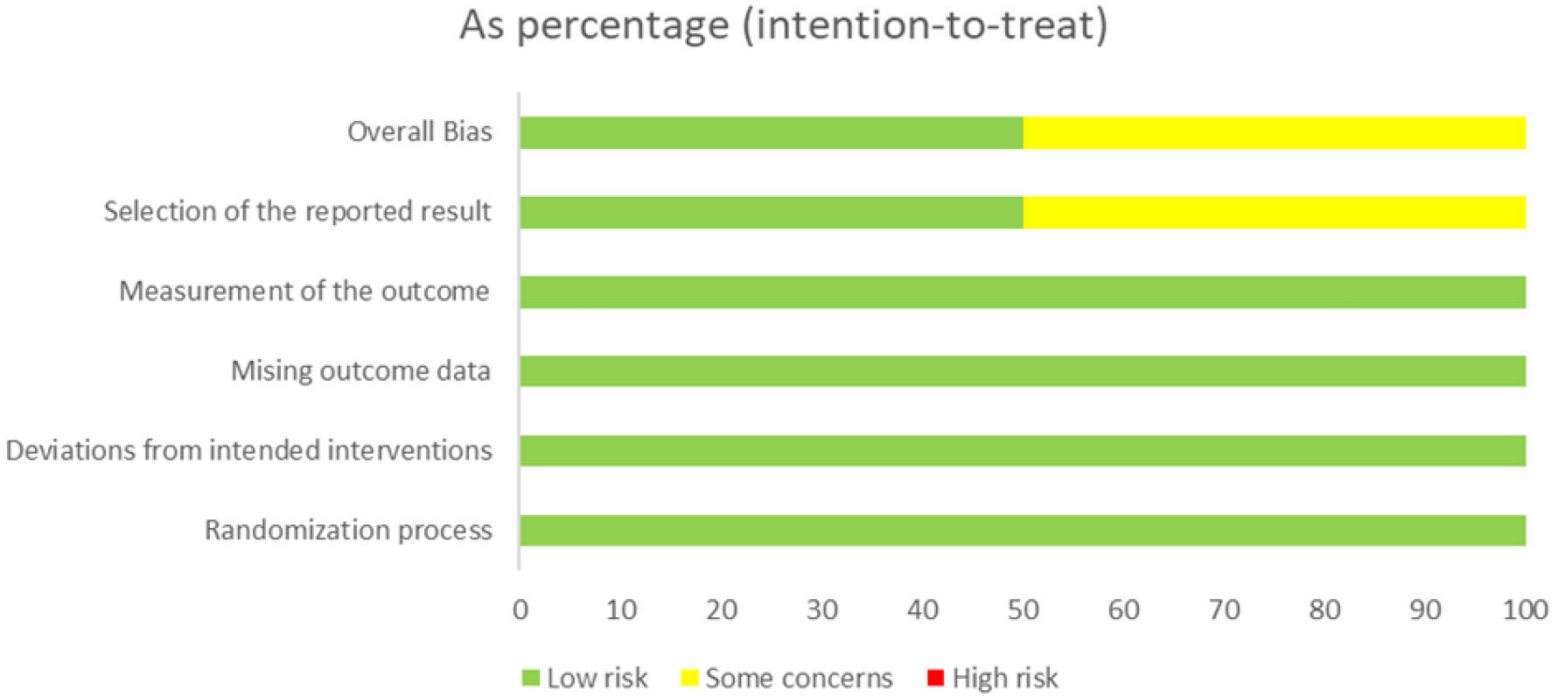
Risk of Bias (RoB 2.0) summary plot. Aggregate summary of risk of bias across all randomized controlled trials, evaluated using the Cochrane RoB 2.0 tool. Most studies were rated as low risk across key domains: randomization, deviations from interventions, missing data, and outcome measurement. Two studies presented “some concerns” due to incomplete pre-registration or selective reporting, while none were rated as high risk.

**Figure 5:**
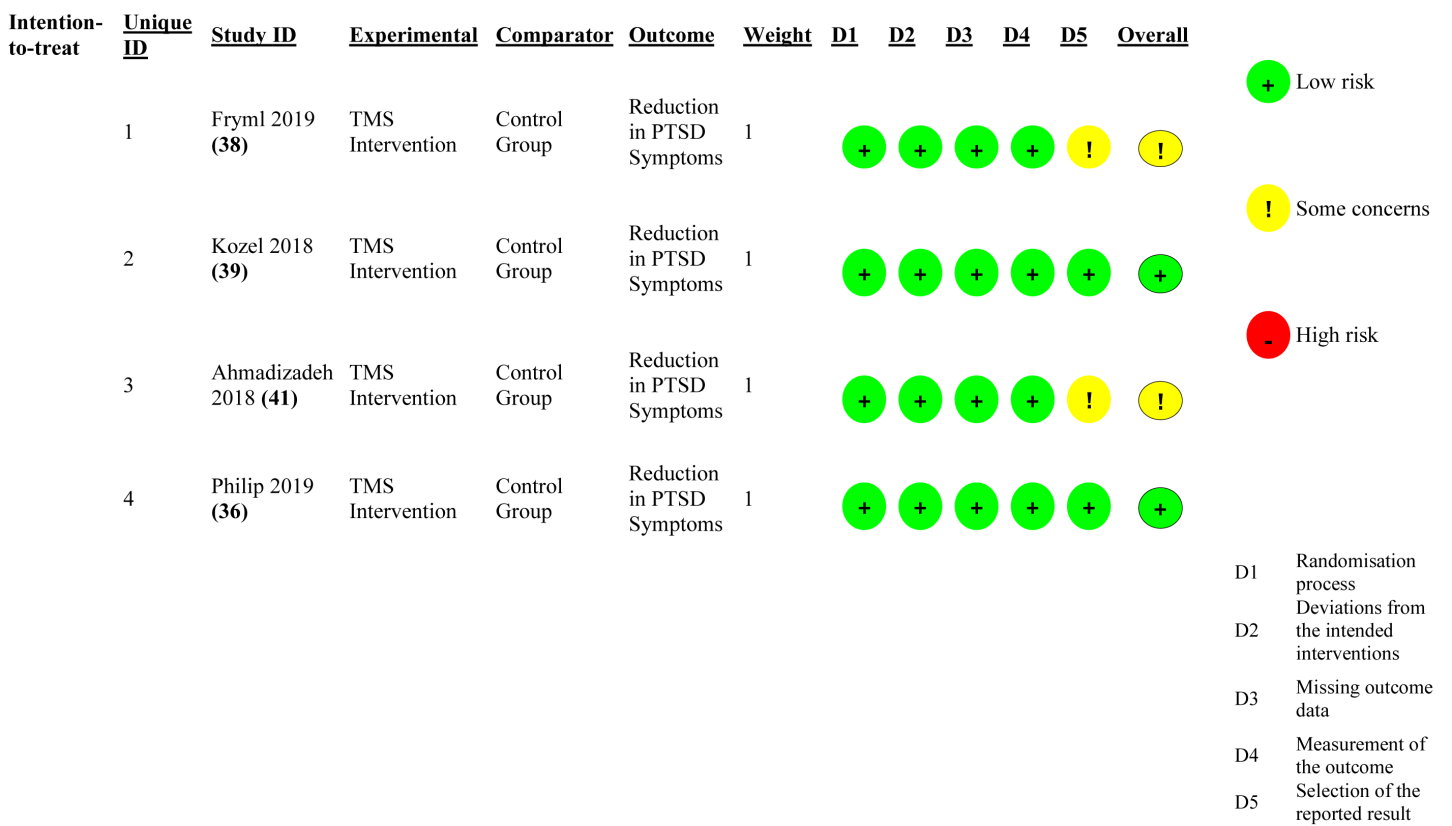
Risk of Bias (RoB 2.0) domain-level visualization. Color-coded assessment of risk of bias for individual studies across RoB 2.0 domains. Green indicates low risk, yellow represents some concerns, and red indicates high risk. No studies demonstrated high risk in any domain, confirming the overall methodological rigor of the included trials.

Risk of bias was evaluated using the Cochrane RoB 2.0 tool (**Figures 4**, **5**). Most randomized trials showed low risk across domains related to randomization, adherence to intervention, completeness of data, and outcome assessment. Two trials presented some concerns regarding selective outcome reporting (37, 40), mainly due to incomplete pre-registration. No studies were judged to be at high risk of bias, suggesting moderate-to-high methodological quality and supporting the internal validity of the pooled results.

### Adverse events and acceptability

TMS was safe and well tolerated across all studies (**Table 3**). The most frequent adverse effects were mild headache (6.4%), scalp discomfort (3.7%), and transient fatigue (2.8%). No serious adverse events were reported. Dropout rates ranged from 5% to 12%, similar between active and sham groups, confirming good acceptability and adherence. These results are consistent with previous large-scale TMS trials for major depression, underscoring the favorable safety profile of TMS in PTSD populations.

**Table 3:** Adverse events and treatment acceptability.

Author (Year)	Total participants	Dropout rate (%)	Common adverse events
Kozel et al. (2018) (39)	32	9.4	Headache (3), scalp discomfort (2)
Fryml et al. (2019) (37)	8	12.5	Mild headache (1)
Kozel et al. (2019) (38)	46	8.6	Fatigue (2), scalp discomfort (1)
Ahmadizadeh et al. (2018) (40)	16	6.3	Headache (1)
Philip et al. (2023) (35)	658	5.1	Mild fatigue (8), scalp discomfort (4)
Overall	760	7.3	Headache, scalp discomfort

Summarizes safety outcomes across included studies, reporting rates and types of adverse events, dropout rates, and the absence of serious adverse effects. Demonstrates overall high tolerability of TMS among combat-related PTSD participants.

## Discussion

Combat-related PTSD may differ meaningfully from civilian PTSD with respect to neurobiological, clinical, and treatment-response characteristics, supporting the need for a dedicated synthesis (16, 24, 34–40). Neuroimaging and neurophysiological studies suggest that military-related PTSD is associated with pronounced alterations in fronto-limbic circuitry, including impaired prefrontal regulatory control over limbic structures involved in threat processing and fear extinction (14–16, 18, 22). Clinically, combat-related PTSD is often characterized by greater symptom chronicity (1, 5, 16), higher severity (14, 16), and elevated rates of comorbid depression, anxiety, and substance use disorders (1–5, 7), which may influence responsiveness to neuromodulation interventions. These factors, together with differences in trauma type, exposure intensity, and baseline neural network dysfunction, may partially account for variability in treatment response observed across PTSD populations (17–19). In the context of an increasingly bellicose global landscape, marked by conventional warfare, non-conventional conflicts, and asymmetric combat scenarios, there is a growing need to identify effective, scalable, and neurobiologically grounded interventions for individuals exposed to combat-related trauma. Consequently, aggregating combat-related and civilian PTSD studies may obscure population-specific effects of TMS, whereas a focused synthesis allows for a more precise evaluation of its therapeutic potential in military and veteran populations. This systematic review and meta-analysis evaluated the efficacy of different transcranial magnetic stimulation (TMS) protocols for combat-related post-traumatic stress disorder (PTSD), responding to the critical need for protocol-level comparisons in military and veteran populations. The current review addressed these limitations by conducting a comprehensive multi-database search, applying PRISMA 2020 and Cochrane standards, and prospectively registering the protocol.

### Interpretation of main findings

Within-group analyses showed a robust pooled reduction in PTSD symptoms (MRAW −20.39), indicating that TMS across diverse protocols produces clinically meaningful improvement in combat-exposed populations. This effect was consistent, with the largest changes observed in high-frequency left-DLPFC rTMS programs (34, 35) and in right-sided 1 Hz inhibitory stimulation (24). These findings support the clinical utility of TMS for combat-related PTSD.

However, the between-group comparison across RCTs did not achieve statistical significance (MD −3.83; p = 0.098). Several methodological factors likely contributed to this discrepancy. First, the RCT sample was small (n = 116 across all trials), limiting the power to detect differences between active and sham stimulation. Second, sham control designs varied markedly, including coil angulation, inactive coils, or low-intensity electrical scalp stimulation, each with different sensory characteristics. Third, the follow-up periods were short (3–8 weeks), potentially underestimating delayed clinical effects. Despite these issues, the direction of effect consistently favored active stimulation, and sensitivity analyses demonstrated that results were stable, suggesting that true differences may have been obscured by methodological constraints.

Subgroup analyses provided additional insight: rTMS showed a significant pooled benefit (MD −12.0), whereas dTMS did not, but the dTMS analysis was based on a single small RCT (n = 8) with wide confidence intervals. This finding therefore reflects inadequate evidence rather than evidence of inefficacy. No iTBS or accelerated TMS trials were eligible for between-group pooling, underscoring the scarcity of comparative data for newer protocols. Thus, the primary conclusion is that insufficient evidence exists to determine superiority among TMS modalities, consistent with the study’s objective.

### Methodological implications of the search strategy and inclusion criteria

The multi-database search strategy and the use of broad TMS-related terms ensured capture of all relevant TMS modalities including rTMS, TBS, dTMS, sTMS, and aTMS across military populations globally. By restricting inclusion to combat-related PTSD diagnosed using DSM-IV, DSM-5, ICD-10, or ICD-11 criteria and requiring validated outcomes (CAPS, PCL-5), the review minimized diagnostic misclassification and ensured psychometric comparability across studies.

The exclusion of civilian PTSD studies, although reducing generalizability, eliminated confounding associated with different trauma etiologies. Including only studies with sham or active comparators strengthened internal validity but reduced the number of eligible trials, especially for newer modalities.

Quality assessment using RoB2 demonstrated low risk across most domains, with only two studies showing “some concerns” due to incomplete preregistration, supporting adequate methodological rigor among included trials. Importantly, no studies were rated as high risk of bias, lending confidence to the internal validity of pooled estimates.

### Interpretation in the context of TMS protocol characteristics

Results suggest that treatment efficacy is less dependent on modality (rTMS, iTBS, or dTMS) than on fundamental parameters such as:

1. target location (left DLPFC most common and effective),
2. stimulation frequency (10 Hz excitatory vs 1 Hz inhibitory),
3. treatment dose (≥20 sessions), and
4. sample characteristics (e.g., chronicity, comorbid mild traumatic brain injury).

The dominance of left-DLPFC 10 Hz rTMS in the pooled dataset representing both the largest sample size and the most consistent outcomes makes it difficult to infer whether other modalities could achieve equivalent or superior efficacy if comparably studied.

Transcranial magnetic stimulation (TMS) is a non-invasive neuromodulation technique that uses a rapidly changing magnetic field generated by a coil placed on the scalp to induce an electric field in cortical tissue, thereby depolarizing excitable neural elements and engaging local microcircuits as well as distributed cortico-cortical and cortico-subcortical networks (41, 42). Repetitive TMS (rTMS) delivers trains of pulses to produce longer-lasting, frequency- and pattern-dependent changes in cortical excitability that are commonly interpreted as LTP/LTD-like plasticity at the systems level (43, 44). Key protocol parameters include (i) stimulation frequency and pattern (e.g., inhibitory low-frequency protocols such as ~1 Hz vs excitatory high-frequency protocols such as 10–20 Hz; as well as patterned approaches such as theta burst stimulation [TBS]), (ii) anatomical target (most commonly prefrontal regions in psychiatric indications), (iii) intensity (typically expressed as a percentage of the individual’s motor threshold to partially control for inter-individual differences in scalp-to-cortex distance and excitability), and (iv) “dose” (operationalized by the number of pulses per session, number of sessions, and inter-train interval), with these parameters jointly determining the magnitude, direction, and durability of after-effects (45, 46). International safety and application guidelines emphasize that the therapeutic and physiological effects of rTMS are inseparable from these parameter choices, because frequency/pattern, intensity, and cumulative pulse dose interact to shape both efficacy and tolerability, underscoring the importance of protocol-level characterization in comparative syntheses (47).

Contemporary neurocircuit models of PTSD consistently implicate dysregulation within fronto-limbic systems that support threat appraisal, fear learning/extinction, salience processing, and top-down emotion regulation, with converging evidence for reduced regulatory control by prefrontal regions over limbic structures such as the amygdala and related nodes (20, 45). This framework provides a mechanistic rationale for why rTMS parameterization (frequency/pattern, target, intensity, and dose) could matter clinically in PTSD: targeting prefrontal control regions with excitatory protocols (e.g., high-frequency rTMS or excitatory TBS patterns) is hypothesized to strengthen prefrontal network engagement and improve downstream regulation of limbic reactivity, whereas inhibitory protocols may be leveraged to dampen maladaptive hyperactivity in specific cortical nodes, depending on the chosen target and symptom-neurocircuit mapping (44, 45). At the network level, human neuroimaging evidence demonstrates that rTMS can bidirectionally modulate prefrontal–amygdala functional coupling as a function of stimulation frequency (e.g., decreased vs increased connectivity following low- vs high-frequency stimulation), supporting the central premise that protocol parameters can translate into measurable shifts in prefrontal–limbic coherence.10 Together, these observations align the protocol-comparison aim of this review with a biologically plausible pathway in which parameter-specific prefrontal stimulation alters fronto-limbic dynamics that are core to PTSD pathophysiology, thereby motivating a careful synthesis of outcomes by stimulation modality and protocol characteristics (48).

### Comparison with existing literature

The present meta-analysis, restricted to combat-related PTSD in military personnel and veterans, found a large pooled within-group reduction in PTSD symptoms after TMS (MRAW ≈ −20 points on CAPS/PCL-5) but a small, statistically non-significant advantage over sham in pooled between-group analyses (MD −3.83; 95% CI −16.32 to 8.65), with moderate heterogeneity and only three RCTs contributing. This pattern is broadly consistent with the trend in the most recent high-quality evidence, which has generally revised earlier, more optimistic estimates downward and emphasized heterogeneity and imprecision.

### Alignment with recent PTSD-specific systematic reviews and meta-analyses

The 2024 Cochrane review by Brown et al. pooled 13 RCTs (n = 577) of rTMS versus sham in adults with PTSD and concluded that active rTMS probably makes little to no difference in PTSD severity immediately post-treatment compared with sham (SMD −0.14; 95% CI −0.54 to 0.27; 3 trials, 99 participants), with moderate certainty and notable heterogeneity once more trials were included in sensitivity analyses (24). This is highly convergent with our between-group estimate: both analyses show substantial within-group improvement but only small, imprecise differences versus sham, and both are limited by few RCTs and wide confidence intervals.

Wang et al. (2025) performed a recent meta-analysis of non-invasive brain stimulation in PTSD, focusing on rTMS and tDCS. They reported global symptom reductions with active NIBS, but effect sizes versus sham were modest and heterogeneous, with better performance in protocols targeting the DLPFC and combining neuromodulation with psychotherapy (49). Our results, which show clinically meaningful pre–post reductions but no clear superiority over control at the aggregate level, are consistent with this pattern. Importantly, our restriction to combat-related PTSD in veterans, and inclusion of large veteran-focused cohorts (e.g., Philip et al., 2023, n = 658), extends the evidence summarized by Brown et al. and Wang et al., which included both military and civilian populations but had fewer or smaller veteran-specific samples (50).

Several recent reviews focusing specifically on TMS or NIBS for PTSD converge on similar conclusions. Cox et al. (2022) synthesized RCTs of different rTMS frequencies and concluded that high-frequency stimulation over prefrontal targets produced small-to-moderate improvements versus sham, but with low-to-moderate certainty because of small sample sizes and heterogeneous protocols (51). Saccenti et al. (2024) performed a systematic review of non-invasive brain stimulation in PTSD and highlighted that, although high-frequency rTMS and theta-burst stimulation (TBS) frequently yielded symptom reductions, the evidence base remained fragmented, with variability in trauma type, comorbidities, and stimulation parameters limiting firm protocol recommendations (52). Liu et al. (2024) similarly pooled rTMS, tDCS, and other neuromodulation modalities and found overall beneficial effects but emphasized substantial heterogeneity and generally small RCTs (53).

Our findings are more conservative than earlier meta-analyses predating 2022, such as Kan et al. (2020) (54), Trevizol et al. (2016) (55), and Berlim et al. (2014) (21), which reported larger standardized mean differences in favor of rTMS versus sham. This discrepancy likely reflects the incorporation of newer neutral or weakly positive trials (including larger, more pragmatic studies and trials with rigorous sham and adjunctive psychotherapy in both arms) into the recent evidence base. The Cochrane analysis by Brown et al. explicitly notes that earlier pooled estimates were inflated by small, early trials and that adding more recent RCTs attenuated the effect size and introduced visible heterogeneity (24). Our results, particularly the non-significant between-group effect, fit within this newer, more conservative estimation landscape.

### Comparison at the level of clinical populations and trauma type

Most recent systematic reviews and meta-analyses have combined civilian and military samples. Brown et al. highlight that many included trials are weighted toward either male veterans or female civilians, and that this mixture may affect generalizability (24). Wang et al. and Saccenti et al. also include heterogeneous trauma types (combat, interpersonal, accidents, disasters) and a mix of primary PTSD versus PTSD comorbid with MDD (49, 52).

By contrast, our meta-analysis is restricted to combat-related PTSD in active-duty military and veterans. The magnitude of within-group change (≈ 20 points on CAPS/PCL-5) is comparable to or slightly larger than pre–post changes reported in mixed-population reviews, where reductions in the range of 10–20 points are typical for active TMS conditions (50). However, the lack of a statistically significant advantage over sham in our pooled between-group analysis suggests that, at least in combat populations, non-specific factors (placebo response, natural fluctuation, concurrent psychotherapy/pharmacotherapy) may account for a substantial portion of the symptomatic improvement. This interpretation is consistent with Cochrane’s finding that rTMS probably has little to no effect versus sham at end of treatment but is associated with considerable heterogeneity, some of which may be population-specific (24).

Our focus on veterans aligns with real-world clinical reports from the US Veterans Health Administration, which describe robust pre–post improvements in both depression and PTSD symptoms with standard left-DLPFC rTMS in veterans, including those with comorbid mild traumatic brain injury, but without controlled comparisons to sham (56). Those naturalistic findings, like our within-group estimates, support clinical utility but cannot disentangle specific from non-specific effects. In this respect, our study fills a gap between controlled but underpowered RCTs and large uncontrolled clinical cohorts by aggregating both RCTs and open-label data in a strictly combat-related sample.

### Comparison across TMS protocols (rTMS, TBS, deep TMS and other variants)

Recent work has increasingly differentiated between conventional high-frequency rTMS, iTBS, deep TMS (dTMS), and combined or accelerated protocols. Wang et al. and Saccenti et al. report that most PTSD evidence still centers on rTMS targeting DLPFC, with fewer trials of iTBS, dTMS or synchronized TMS. When TBS is examined, studies often show within-group improvements of similar magnitude to conventional rTMS but without clear superiority in head-to-head or sham-controlled designs (57, 58).

Our results mirror this landscape. In subgroup analysis, we observed a trend toward benefit for conventional rTMS and no clear effect for dTMS, with wide confidence intervals reflecting the very small dTMS sample (one 8-patient RCT). The direction of effects is compatible with randomized pilot trials of deep TMS combined with brief exposure, which showed promising symptom reductions but failed to reach statistical significance in small samples (58). Intermittent TBS, represented in our dataset by a small pilot in Australian veterans and prior work in US veterans (e.g., Philip et al., (23, 27), has shown clinically meaningful pre–post improvements and reasonable one-year durability but limited superiority to sham and no clear advantages over standard rTMS in the limited direct comparisons available (59).

This is consistent with broader neuromodulation meta-analyses across psychiatric disorders, which typically find that iTBS is non-inferior to standard rTMS for depression but not clearly superior, and that protocol choice may be driven more by logistical efficiency than by established efficacy differences (60, 61). Our meta-analysis does not provide evidence that any specific protocol (rTMS, iTBS, or dTMS) is definitively superior for combat-related PTSD, which aligns with the cautious stance taken by recent reviews.

### Methodological comparison and risk of bias

Methodologically, our review followed PRISMA 2020 and Cochrane guidance, was prospectively registered in PROSPERO, and used RoB 2.0 for RCTs, mirroring best practices adopted in Brown et al. (Cochrane) (24), Saccenti et al. (Brain Sci) (52), and Wang et al. (49). Like these reviews, we applied random-effects models and quantified heterogeneity with I², which was high in our within-group analysis (≈ 89%), comparable to the substantial heterogeneity reported in recent PTSD-focused NIBS meta-analyses (53, 54).

Our inclusion of open-label and pilot studies in the within-group analysis is similar to the approach taken by Liu et al. and some earlier meta-analyses, which expanded beyond strictly sham-controlled RCTs to increase ecological validity (53, 54). However, our between-group analysis was restricted to randomized, sham-controlled trials, aligning with Cochrane and Cox et al. (51) This separation of within- and between-group effects is important, as it highlights that large pre–post changes do not automatically imply strong specific effects versus sham.

Compared with some earlier work, our risk-of-bias profile appears slightly more favorable: no RCTs were rated as high risk of bias, whereas Brown et al. reported several RCTs with high risk, particularly regarding adverse event reporting and selective outcomes (24). This difference largely reflects our more recent evidence base (which includes newer, better-reported trials) and our focus on a subset of studies with more complete data for meta-analysis. Nonetheless, small sample sizes in several included RCTs (especially for dTMS and iTBS) mean that imprecision remains a major limitation shared with the wider literature.

### Statistical and clinical magnitude of effects

From a statistical standpoint, our non-significant pooled MD versus sham and wide confidence intervals closely parallel the Cochrane estimate (SMD −0.14; 95% CI −0.54 to 0.27) (24). More optimistic estimates in some 2022–2024 reviews (49–53) often arise from including more diverse NIBS modalities, combining change and endpoint data, or pooling across broader populations and settings. When considered in this context, our study supports the emerging consensus that:

1. TMS (particularly rTMS over DLPFC) is associated with clinically meaningful symptom reductions within treated groups.
2. The specific advantage over sham is small-to-moderate at best and not consistently statistically significant across RCTs.
3. Effect sizes are highly protocol- and sample-dependent, with considerable between-study heterogeneity.

Clinically, a ≈ 20-point reduction on CAPS or PCL-5 is generally considered a large, meaningful improvement for individual patients, and our pooled within-group estimate is typical or slightly greater than that reported in other recent analyses (62). However, because control conditions also tend to improve (e.g., trauma-focused psychotherapy, pharmacotherapy, and expectancy/placebo effects), the net incremental benefit attributable to TMS remains uncertain, especially in highly treated, chronic veteran populations.

### Position relative to broader neuromodulation and guideline literature

Beyond PTSD-focused reviews, several recent umbrella and scoping reviews of neuromodulation across mental disorders, like Hyde et al., 2022 (63), Trapp et al., 2024 (60) characterize the PTSD rTMS evidence as promising but preliminary, with insufficient high-quality trials to support strong recommendations. UpToDate’s 2025 PTSD treatment overview similarly frames rTMS as an experimental or second-line option rather than a standard of care (64). Our results reinforce this cautious positioning: despite robust within-group change and an excellent safety profile, the absence of a clear, consistent between-group advantage over sham in combat veterans argues against considering any TMS protocol as a fully established first-line treatment for combat-related PTSD.

At the same time, the safety data in our review (no serious adverse events, low dropout rates) align with the safety profile reported across PTSD-specific and cross-diagnostic TMS literature, which consistently finds low rates of serious adverse events and good tolerability. Together, this suggests that TMS is a rational component of multimodal care for combat-related PTSD, particularly when delivered in specialized settings, but that definitive protocol optimization and robust evidence of superiority over sham remain key targets for future research.

### Safety and acceptability

Across all studies, TMS demonstrated an excellent safety profile. No serious adverse events were reported, and dropout rates were low (overall 7.3%), comparable between active and sham conditions. Adverse events primarily mild headache, scalp discomfort, and transient fatigue were consistent with known TMS safety profiles. This supports TMS as a feasible and acceptable treatment option for veterans, including those with comorbid mild traumatic brain injury.

### Limitations

Despite these strengths, several limitations warrant consideration. The small number of RCTs and limited sample sizes restricted power to detect between-protocol differences. Heterogeneity across TMS parameters, frequency, intensity, target site, session count, and sham design, complicated pooled estimates. Follow-up durations were uniformly short, precluding assessment of long-term durability except in isolated studies. Newer modalities such as EEG-guided TMS, accelerated TMS, and synchronized TMS lacked sufficient controlled data for inclusion. Additionally, the sample was predominantly male, reflecting military demographics but limiting generalizability to female veterans.

### Clinical implications

rTMS, particularly left-DLPFC 10 Hz protocols, currently possesses the strongest evidence base and represents a reasonable first-line neuromodulation option for combat-related PTSD. iTBS and accelerated TMS offer practical advantages, including shorter treatment durations, but require more robust comparative trials. EEG-guided personalized TMS shows promise but remains investigational. Clinicians should interpret protocol differences with caution, as the current evidence base is insufficient to recommend one modality over another.

### Future research

Future research on TMS for PTSD should prioritize large, adequately powered head-to-head randomized controlled trials comparing rTMS, iTBS, dTMS, and personalized TMS, especially using accelerated or short protocols while employing standardized stimulation parameters and harmonized PTSD outcome measures. Studies should include long-term follow-up of 12 to 24 months to evaluate durability of effects and functional recovery, alongside mechanistic investigations that integrate neuroimaging and electrophysiology to clarify neural pathways of response. Research must also examine key moderators such as sex, age, illness chronicity, and comorbid mild traumatic brain injury, and incorporate rigorously standardized sham conditions with validated blinding to ensure methodological integrity.

## Conclusion

In summary, TMS produced a large within-group reduction in PTSD symptoms, with a pooled mean change of −20.39 points (95% CI −23.94 to −16.83) across 963 combat-exposed participants. This improvement was consistent across studies and protocols, indicating a robust clinical effect. However, the analysis also showed substantial heterogeneity (I² = 88.7%), and randomized between-group comparisons did not demonstrate a statistically significant advantage of active TMS over sham. No TMS modality rTMS, iTBS, or dTMS showed clear superiority, largely due to small sample sizes, methodological variability, and limited controlled data, especially in newer protocols. Safety outcomes were favorable, with no serious adverse events reported.

Overall, while TMS is clinically beneficial and well tolerated in combat-related PTSD, current evidence does not justify preferential selection of any specific stimulation protocol. Adequately powered, head-to-head randomized trials with standardized parameters and longer follow-up are required to determine whether any modality offers a meaningful therapeutic advantage in this population.

## Data Availability

The raw data supporting the conclusions of this article will be made available by the authors, without undue reservation.

## Author’s Note

The study was presented at the 25th World Congress of Psychiatry in Prague, Czech Republic, under the title: “Efficacy of Transcranial Magnetic Stimulation in the Treatment of Combat-Related PTSD: A Systematic Review” (ID 3932).
